# Supplementary material for: Lactate dehydrogenase A-coupled NAD+ regeneration is critical for acute myeloid leukemia cell survival
Source: Cancer Metab. 2025 May 19;13:22. doi: 10.1186/s40170-025-00392-4 (PMC12090514; doi:10.1186/s40170-025-00392-4)
Supplement: Supplementary file 1 — Supplementary Material 1 [file 40170_2025_392_MOESM1_ESM.pdf]

LACTATE DEHYDROGENASE A-COUPLED NAD+ REGENERATION IS CRITICAL FOR ACUTE MYELOID LEUKEMIA CELL SURVIVAL

Ayşegül Erdem[1], Séléna Kaye[1], Francesco Caligiore[2], Manuel Johanns[3], Fleur Leguay[1], Jan Jacob Schuringa[4], Keisuke Ito[5], Guido Bommer[2] and Nick van Gastel[1,6]

[1]Cellular Metabolism and Microenvironment Laboratory, de Duve Institute, UCLouvain, Brussels, Belgium. [2]Biochemistry and Metabolic Research Group, de Duve Institute, UCLouvain, Brussels, Belgium. [3]Protein Phosphorylation Group, de Duve Institute, UCLouvain, Brussels, Belgium. [4]Department of Experimental Hematology, University Medical Center Groningen, University of Groningen, Groningen, The Netherlands. [5]Ruth L. and David S. Gottesman Institute for Stem Cell and Regenerative Medicine Research, Albert Einstein College of Medicine, Bronx, NY 10461, USA; Department of Cell Biology, Albert Einstein College of Medicine, Bronx, NY 10461, USA; Departments of Oncology and Medicine, Albert Einstein College of Medicine-Montefiore Health System, Bronx, NY 10461, USA. [6]WELBIO Department, WEL Research Institute, Wavre, Belgium.

Supplementary Material

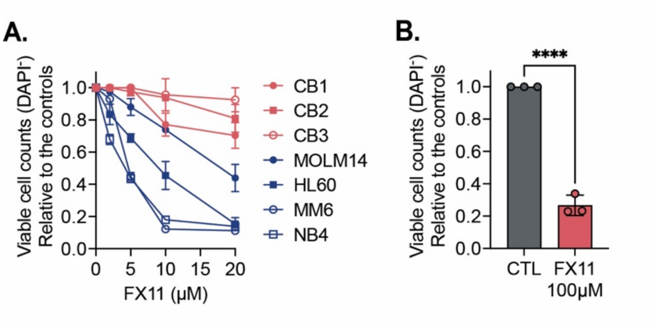

Supplementary Figure 1.

A. Number of viable (DAPI^{-}) cells relative to controls in AML cell lines and healthy cord blood. (CB)-derived CD34^{+} cells following treatment with increasing doses of FX11 for 48 hours. Each dot represents the mean of biological triplicates. **B.** Number of viable (DAPI^{-}) cells relative to controls in healthy cord blood-derived CD34^{+} cells following 100 μM FX11 treatment for 48 hours. Each dot represents the mean of biological triplicates. Lines and error bars represent mean ± SEM. **B**: Student's t test; ****$p < 0.0001$.

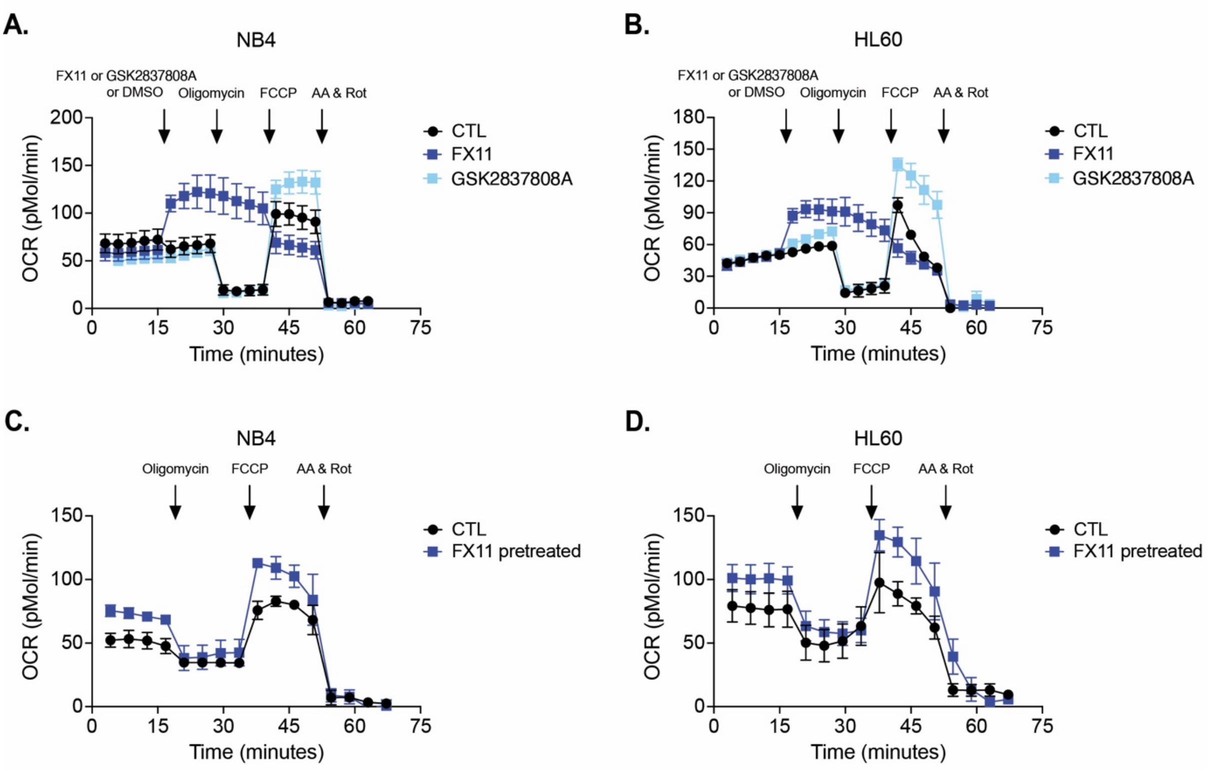

Supplementary Figure 2.

A-B. Real-time oxygen consumption rate (OCR) of AML cell lines NB4 (A) and HL60 (B) (mean ± SEM of biological triplicates) measured by Seahorse bioassay after sequential injections of either of the two LDHA inhibitors (20 μM of FX11, 10 μM of GSK2837808A), oligomycin, FCCP and antimycin a (AA) & rotenone (Rot). **C-D**. Real-time OCR measured by Seahorse bioassay of AML cell lines NB4 (C) and HL60 (D) (mean ± SEM of biological quadruplicates) pretreated for 24 hours with 10 μM FX11 or vehicle, followed by sequential injections of oligomycin, FCCP and AA & Rot.

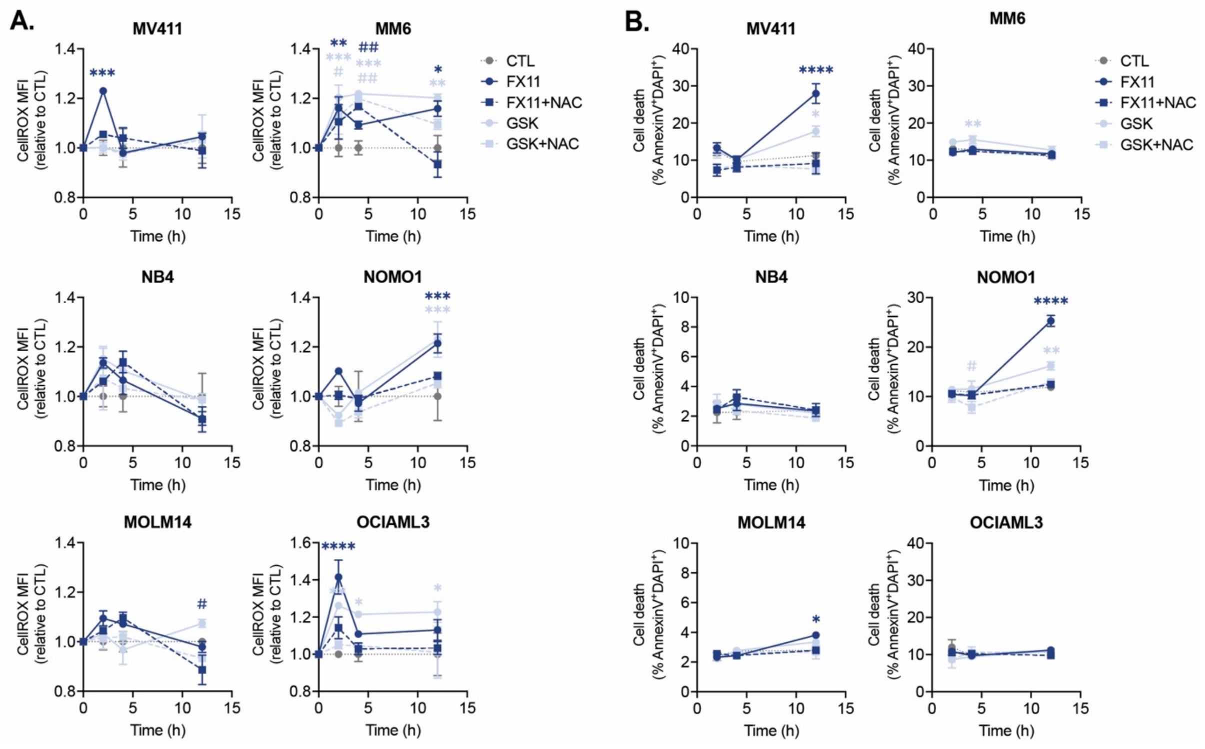

Supplementary Figure 3.

A. Fold change of CellROX Orange mean fluorescent intensity (MFI) in AML cell lines exposed to 20 μM FX11 or 10 μM GSK2837808A in the presence and absence of NAC (N-acetyl-L-cysteine, 2 mM) for different durations. **B**. Percentage dead (Annexin $V^{+}DAPI^{+}$) AML cells in cultures exposed to 20 μM FX11 or 10 μM GSK2837808A in the presence and absence of NAC (N-acetyl-L-cysteine, 2 mM) for different durations. **A,B**: one-way ANOVA for multiple comparisons. All experiments: dots and error bars represent mean ± SEM of biological replicates measured in technical triplicate. $^{*}p < 0.05$; $^{**}p < 0.01$; $^{***}p < 0.001$; $^{****}p < 0.0001$ for FX11 (dark blue) or GSK2837808A (light blue) versus control, $^{\#}p < 0.05$; $^{\#\#}p < 0.01$ for FX11 (dark blue) or GSK2837808A (light blue) versus control.

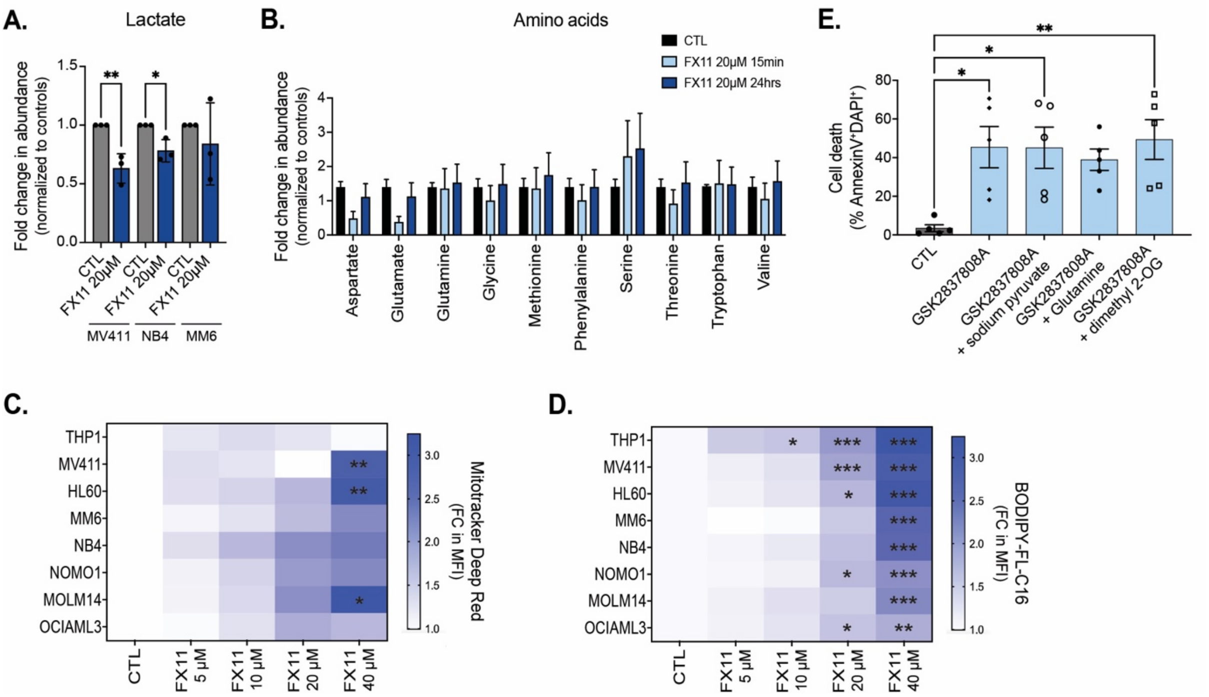

Supplementary Figure 4.

A. Intracellular lactate levels in different AML cell lines after 24 hours of treatment with 20 μM FX11, as measured by LC-MS. Each dot represents a technical replicate. **B.** Relative abundance of amino acids in NB4 cells after 15 minutes and 24 hours of treatment with 20 μM FX11, as measured by LC-MS. Bars show mean ± SEM of biological triplicates. **C.** Heatmap (mean of biological triplicates) showing the fold change of MitoTracker Deep Red mean fluorescent intensity in AML cell lines exposed to increasing concentrations of FX11 for 24 hours, compared to DMSO treated controls. **D.** Heatmap (mean of biological triplicates) showing the fold change of BODIPY-FL-C_{16} mean fluorescent intensity in AML cell lines exposed to increasing concentrations of FX11 for 24 hours, compared to DMSO treated controls. **E.** Percentage dead (Annexin $V^{+}DAPI^{+}$) AML cells treated with 20 μM GSK2837808A alone or with 2 mM sodium pyruvate, 2 mM glutamine or 400 μM dimethyl 2-OG (2-oxoglutarate) for 24 hours. Each dot represents a distinct AML cell line, as mean of biological triplicates. **A**: Student's t test; **B,C,D,E**: one-way ANOVA for multiple comparisons. All experiments: bars and error bars represent mean ± SEM. $*p < 0.05$; $**p < 0.01$; $***p < 0.001$.

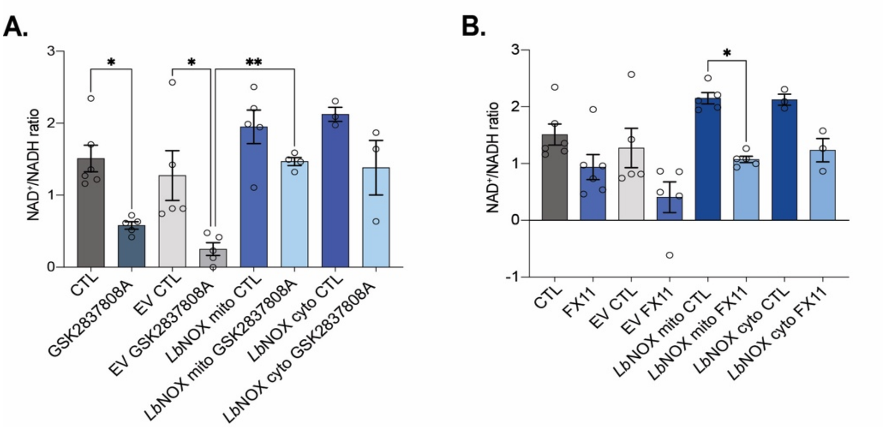

Supplementary Figure 5.

A-B. Cellular NAD+/NADH ratio in NB4 control cells, NB4 cells transduced with an empty vector (EV) and NB4 cells overexpressing a mitochondrial or cytosolic variant of *Lb*NOX, treated with vehicle or 20 μM GSK2837808A (A) or 20 μM FX11 (B) for 24 hours, as measured by enzymatic assay. **A,B**: one-way ANOVA for multiple comparisons. All experiments: bars and error bars represent mean ± SEM. *$p < 0.05$; **$p < 0.01$.

Supplementary Table 1: Patient characteristics

Sample ID	Sample information & known mutations
AML1	BCORL1, DNMT3A, NRAS, PTPN11, TET2, U2AF1I
AML2	FLT3-ITD, NPM1, DNMT3A, TP53
AML3	MLL rearrangement
AML4	BCOR, DNMT3A, NRAS, RUNX1, STAG2
AML5	AML (no $CD34^+$ cells)
AML6	KIT, PTPN11, WT1
AML7	NPM1, TET2
AML8	ASXL1 germline
AML9	Chronic neutrophilic leukemia (CNL) with classic activating mutation in CSF3R (CSF3R-T618I).
AML10	Venetoclax resistant AML; FLT3, IDH2, RUNX1, TET2
AML11	FLT3-ITD, NPM1, WT1 frameshift
AML12	DNMT3A, NPM1, WT1
AML13	BRAF, GNAS, JAK2, NRAS, TET2
AML14	DNMT3A, FLT3, NPM1
AML15	CEBPA
